# Ethnic and socioeconomic trends in breast cancer incidence in New Zealand

**DOI:** 10.1186/1471-2407-10-674

**Published:** 2010-12-07

**Authors:** Ruth Cunningham, Caroline Shaw, Tony Blakely, June Atkinson, Diana Sarfati

**Affiliations:** 1 Department of Public Health, University of Otago Wellington, PO Box 7343, Wellington 6242, New Zealand

## Abstract

**Background:**

Breast cancer incidence varies between social groups, but differences have not been thoroughly examined in New Zealand. The objectives of this study are to determine whether trends in breast cancer incidence varied by ethnicity and socioeconomic position between 1981 and 2004 in New Zealand, and to assess possible risk factor explanations.

**Methods:**

Five cohorts of the entire New Zealand population for 1981-86, 1986-1991, 1991-1996, 1996-2001, and 2001-2004 were created, and probabilistically linked to cancer registry records, allowing direct determination of ethnic and socioeconomic trends in breast cancer incidence.

**Results:**

Breast cancer rates increased across all ethnic and socioeconomic groups between 1981 and 2004. Māori women consistently had the highest age standardised rates, and the difference between Māori and European/Other women increased from 7% in 1981-6 to 24% in 2001-4. Pacific and Asian women had consistently lower rates of breast cancer than European/Other women over the time period studied (12% and 28% lower respectively when pooled over time), although young Pacific women had slightly higher incidence rates than young European/other women. A gradient between high and low income women was evident, with high income women having breast cancer rates approximately 10% higher and this difference did not change significantly over time.

**Conclusions:**

Differences in breast cancer incidence between European and Pacific women and between socioeconomic groups are explicable in terms of known risk factors. However no straightforward explanation for the relatively high incidence amongst Māori is apparent. Further research to explore high Māori breast cancer rates may contribute to reducing the burden of breast cancer amongst Māori women, as well as improving our understanding of the aetiology of breast cancer.

## Background

Breast cancer is the most commonly diagnosed non-skin cancer and the leading cause of cancer death for New Zealand women [1]. Rates of breast cancer in New Zealand are similar to other developed countries, with an age-standardised rate of around 80 per 100,000 women [2]. The incidence of breast cancer has been increasing in New Zealand, with rates nearly doubling in the second half of last century. This increase is likely to be due to changes in both risk factor patterns and methods of diagnosis [3,4].

Breast cancer incidence varies between social groups. In general groups with higher income and education have higher rates of breast cancer [3,4]. White ethnic groups tend to have higher rates of breast cancer than non-white groups in the same areas [5-7]. These differences in rates are thought to relate to differences in risk factor distribution between socioeconomic and ethnic groups [8-10].

Epidemiological studies suggest that reproductive factors play a major role in determining breast cancer risk. Older age at first birth, lower parity, lack of breast feeding, younger age at menarche, older age at menopause and use of menopausal hormone therapy (HT) are all related to increases in breast cancer risk [11], and can all vary between social groups [12]. Other important modifiable risk factors that may be relevant to differences between social groups include physical inactivity, post menopausal obesity, and regular alcohol consumption [11]. Environmental and genetic differences between groups may also be important. Breast cancer screening will also increase incidence [13,14], and may contribute to differences between groups where there are differences in screening coverage.

Differences in breast cancer incidence between social groups vary by age [15,16]. This is likely to relate to variations in risk factor distribution in social groups over time (a cohort effect) as well as certain risk factors having a differential effect by age. Two risk factors for which this is the case are childbearing and obesity [17,18]. It appears that childbearing has an initial cancer promoting effect, and then a later protective effect, so that young age at first birth initially increases breast cancer risk above childless peers, but then reduces breast cancer risk in later life [8,19]. Obesity reduces breast cancer risk pre-menopausally, but increases breast cancer risk following menopause [20].

This paper examines variation in breast cancer incidence by socioeconomic status and ethnicity in New Zealand over 25 years from 1981. There are a number of reasons for investigating this issue. Firstly, there are known differences between social groups in New Zealand in terms of risk factor patterns and screening, and so differences in breast cancer incidence between groups are expected. Moreover, reproductive patterns and other important risk factors are changing over time and differentially between socioeconomic and ethnic groups. We would therefore expect that ethnic and socioeconomic patterns in breast cancer incidence may also be changing over time. These differences may also vary with age, as they do between social groups in other countries. Secondly, "expected" patterns of breast cancer incidence between social groups based on risk factor distribution are not always seen, pointing to alternative aetiological explanations [21,22]. In New Zealand, Māori (the Indigenous population) women have higher parity and earlier childbearing than European women, a pattern which would perhaps predict a lower incidence of breast cancer, and yet earlier studies have found that Māori women have a similar incidence of breast cancer to New Zealand European women (who have similar rates of breast cancer to women in other developed countries) [23-25]. It is therefore particularly important to examine ethnic trends in New Zealand, to further elucidate the unexpectedly high rates amongst Māori women when compared to European women.

Thirdly, recent work in New Zealand suggests an emerging trend of higher breast cancer mortality among Māori women and women of lower socioeconomic status [26], but it is difficult to know whether this is due to increasing incidence, decreasing survival, or a combination of the two, without an analysis of incidence (and survival) data over time. The linkage of census and cancer registry data in New Zealand allows a rigorous analysis of socioeconomic and ethnic trends over an entire country and through a 25 year period. This linkage removes the potential for bias occurring when socioeconomic status and ethnicity are measured differently in numerator (usually cancer registry) compared with denominator (usually census) data.

The aims of this paper are:

To examine differences in breast cancer incidence between ethnic and socioeconomic groups

To examine whether differences in incidence between social groups in New Zealand vary over time and by age

## Methods

### Data

The datasets were created through linking records from the New Zealand Cancer Registry (a population based cancer register which collects information on all malignant tumours except basal and squamous cell skin cancers, with mandatory notification since 1994) and records from a 5 yearly census of population and dwellings. Five closed cohorts were created of the New Zealand usual resident population (all ages) on census night 1981, 1986, 1991, 1996, 2001, followed up for incident cancer(s) until the subsequent census or in the case of the 2001 cohort, until 31 December 2004 (the most recent data available at the time of the study's record linkage). Cohorts were created using probabilistic record linkage software (QualityStage) to anonymously link census and cancer register records within a geographic area (meshblock or census area unit) on sex, date of birth, ethnicity, and country of birth. Details on the linkage methods, process and outputs are detailed elsewhere [27,28].

Briefly, 73.2% (1981-86) to 81.7% (2001-04) of eligible cancer registry records were linked to a census record, with 95.2% (1981-86) to 96.9% (2001-04) of these linked census-cancer records estimated to be true links. To adjust for underestimation of rates using the linked datasets, and to correct for any linkage bias whereby the percentage of eligible cancer records linked varied by socio-demographic characteristics, we calculated weights for strata of age, sex, ethnicity, region, small area deprivation, and time since census. All analyses presented in this report use these weights.

Approval was granted for this project under the Statistics New Zealand Data Integration Policy [29], and the Wellington Ethics Committee granted ethics approval for CancerTrends (Ref 04/10/093).

### Variables

#### Exposures

There are four main ethnic groups in New Zealand: 1. the Indigenous people (Māori) who migrated to New Zealand c.1000 AD; 2. those of European origin who commenced migration in the 1800s; 3. people from islands in the Pacific region, with the main migration occurring between 1945 and 1980; and 4. a substantial 'Asian' ethnic group, mostly from a recent wave of migrants [30-32]. By the 2006 census the New Zealand population was just over 4 million, with 65% European, 14% Māori, 7% Pacific, 9% Asian, and around 11% identifying with other ethnic groups [33].

A modified total ethnicity approach was used for this work. Total ethnicity places an individual in all ethnic groups that they identify with, thus capturing (most) multiple ethnic affiliations of individuals [34]. If individuals indicated any/all of Māori, Pacific and/or Asian ethnic affiliation they were placed in any/all of Total Māori, Total Pacific, Total Asian ethnic groups. The residual people who did not indicate any of the above ethnic affiliations were placed in the non-Māori/Pacific/Asian (nMPA). For simplicity this category is referred to as European/Other in this paper. The 1981 census question was based on ethnic origin rather than ethnic affiliation and blood quantum measures were used. In order to convert this into total ethnicity to be consistent with later years, we classified someone as Māori if they recorded any fraction as Māori, and likewise for Pacific and Asian.

**Household equivalised income **was calculated and assigned to each individual within the household. Personal incomes were CPI adjusted to 2001, summed over the household and adjusted for number of individuals within the household, using the New Zealand-specific Jensen equivalisation index [35]. Equivalised incomes were then attached to each individual. In order to create income tertiles for analyses, individuals were grouped into 5 year age groups pooled across cohorts, and then ranked and divided into tertiles within each of these five-year age groups.

Analyses were also performed using education as a measure of socioeconomic position, and are available from the authors on request.

#### Outcome

Cancer outcome was assessed by having a cancer registered on the NZ Cancer Registry. All breast cancers (ICD-10 code C50) were included. Ductal carcinoma in situ (DCIS) was excluded. Cancers prior to 2000 were forward mapped from ICD-9 to ICD-10 codes using mapping codes provided by New Zealand Health Information Service.

### Analyses

For the analyses presented here, data were restricted to adults aged 25 and over who were in their usual residence on census night. All analyses were conducted in SAS v8.2 or v9.

Person time was censored after development of the first breast cancer. Incidence rates, rate ratios and rate differences (and 95% confidence intervals) were calculated after direct standardisation of the cohorts to the age structure of the WHO World Standard population [36], and to the ethnic structure of the 2001 New Zealand population for trends by income (because ethnicity may act as a confounder on the income - breast cancer relationship). All analyses were performed on complete data only, with no imputation of missing data.

Statistical tests of trend were conducted for rates, and rate differences, and of the log transformed rate ratios. All measures were also calculated for all five cohorts pooled [28].

## Results

Table 1 shows the numbers of cancers and total person time, in each of the ethnic and socioeconomic groups in each cohort.

**Table 1:** Number of cancers* and women-years of observation (25 + years) across five time periods according to ethnicity and income

	1981-86		1986-91		1991-96		1996-2001		2001-04	
	Cancers (n)	Person years	Cancers (n)	Person years	Cancers (n)	Person years	Cancers (n)	Person years	Cancers (n)	Person years
**Ethnicity**										
Total Mäori	348	367,781	486	424,739	687	485,574	1,044	628,063	900	484,431
Total Pacific	87	105,369	162	138,460	168	189,060	288	238,991	255	205,681
Total Asian	30	50,527	60	71,662	111	145,207	264	261,012	351	282,592
European/Other	5,229	3,957,509	6,825	4,272,857	7,509	4,472,644	8,748	4,620,738	7,638	3,587,142
Miss Eth	132	70,116	60	60,745	30	45,976	96	70,897	90	52,834
**Income**										
Low Income	1,911	1,404,919	2,235	1,545,090	2,250	1,508,029	2,799	1,582,935	2,523	1,244,643
Medium Income	1,551	1,251,068	2,520	1,533,130	3,063	1,643,443	3,240	1,648,529	2,436	1,107,475
High Income	1,449	1,099,996	1,878	1,098,036	2,115	1,369,292	2,805	1,561,314	2,748	1,353,848
Missing Income	909	790,545	942	783,240	1,065	807,564	1,557	998,890	1,512	887,079

*All count data are random rounded to a near multiple of 3, with a minimum cell size of 6, as per Statistics New Zealand protocol

### Ethnic trends

**Figure 1:**
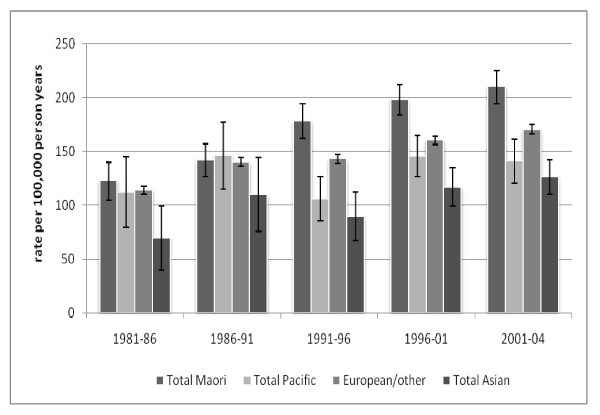
**Trends in age standardized incidence rates of breast cancer by time period and ethnic group, age 25+**.

Figure 1 and Table 2 show age standardised breast cancer rates for Māori, Pacific, Asian and European/other women in each time period. Rates for Māori women increased seventy percent in the time period studied, compared to about 50% for European/other (p for trend both < 0.01). Incidence rates in Pacific and Asian women increased by 25% and 80% respectively.

**Table 2:** Age standardised breast cancer rates, rate ratios and rate differences (compared to European/Other) by ethnicity, age 25+.

Ethnicity	Cohort	SR	95% CI	SRR	95% CI	SRD	95% CI
Total Mäori	1981-86	123	(105 - 140)	1.07	(0.93 - 1.24)	8.4	(-9.2 - 26)
	1986-91	142	(126 - 157)	1.02	(0.91 - 1.14)	2.2	(-14 - 18)
	1991-96	178	(162 - 194)	1.25	(1.13 - 1.37)	35	(18 - 52)
	1996-01	198	(184 - 212)	1.23	(1.14 - 1.33)	38	(23 - 52)
	2001-04	210	(194 - 225)	1.23	(1.14 - 1.33)	39	(23 - 56)
	% Change		71%				
	*P (Trend)*		< .*01*		*0.15*		*0.06*
	Pooled	168	(161 - 175)	1.17	(1.11 - 1.22)	24	(16 - 31)
							
Total Pacific	1981-86	112	(79 - 145)	0.98	(0.73 - 1.32)	-2.0	(-35 - 31)
	1986-91	146	(115 - 177)	1.05	(0.84 - 1.30)	6.6	(-25 - 38)
	1991-96	106	(86 - 126)	0.74	(0.62 - 0.89)	-37	(-57 - -17)
	1996-01	145	(126 - 165)	0.90	(0.79 - 1.04)	-15	(-35 - 4.7)
	2001-04	141	(121 - 161)	0.83	(0.72 - 0.95)	-29	(-50 - -9.2)
	% Change		25%				
	*P (Trend)*		*0.38*		*0.39*		*0.37*
	Pooled	129	(118 - 141)	0.90	(0.82 - 0.98)	-15	(-26 - -3.0)
							
Total Asian	1981-86	69.9	(40.5 - 99.3)	0.61	(0.40 - 0.93)	-44	(-74 - -15)
	1986-91	110	(76 - 144)	0.79	(0.58 - 1.08)	-29	(-64 - 5.1)
	1991-96	89.6	(67.5 - 112)	0.63	(0.49 - 0.80)	-53	(-76 - -31)
	1996-01	117	(99 - 135)	0.73	(0.63 - 0.86)	-43	(-62 - -25)
	2001-04	126	(110 - 142)	0.74	(0.65 - 0.84)	-44	(-61 - -27)
	% Change		80%				
	*P (Trend)*		*0.04*		*0.45*		*0.89*
	Pooled	101	(90 - 113)	0.70	(0.63 - 0.79)	-43	(-54 - -31)
							
European/Other	1981-86	114	(110 - 118)	1		0	
	1986-91	140	(136 - 144)	1		0	
	1991-96	143	(139 - 147)	1		0	
	1996-01	160	(156 - 164)	1		0	
	2001-04	170	(166 - 175)	1		0	
	% Change		49%				
	*P (Trend)*		< .*01*				
	Pooled	144	(142 - 146)				

*p test is test for linear trend

Both the relative and absolute differences between Māori and European/other women increased between 1981 and 2004 (Table 2). Māori women had 7% higher rates than European/other women in 1981-86, and 24% higher rates in 2001-4 (SRR 1.07 in 1981-86 and 1.24 in 2001-04), and the absolute difference increased from 9 to 40 per 100,000 women. By contrast Asian and Pacific women had consistently lower breast cancer rates than European/Other women and the differences between Pacific and Asian and European/Other women did not change markedly over time.

Table 3 shows the patterns of differences between Māori, Pacific and European/Other ethnic groups at different ages, pooled for all cohorts to increases statistical precision and stability when assessing heterogeneity by age. Compared to European/Other women, Māori women had a significant 15-17% increased risk across all age groups. The rate ratio for Pacific women varied with age with young Pacific women having an increased risk (SRR 1.17; 95% CI 1.01-1.35) and older women a decreased risk (SRR 0.77; 95% CI 0.62-0.96) compared to European/Other women. Asian women had a lower risk than European/Other women across all ages, with a decreasing risk by age relative to European/Other women.

**Table 3:** Age-standardised rate ratios (SRR) of breast cancer, by age group, pooled across all cohorts, for Māori, Pacific and Asian compared to European/Other

		25-44 years		45-64 years		65+ years	
Ethnicity		SRR	95% CI	SRR	95% CI	SRR	95% CI
Total Mäori vs European	Pooled	1.16	(1.06 - 1.27)	1.17	(1.10 - 1.24)	1.14	(1.04 - 1.26)
							
Total Pacific vs European	Pooled	1.16	(1.01 - 1.35)	0.94	(0.82 - 1.07)	0.77	(0.62 - 0.96)
							
Total Asian vs European	Pooled	0.86	(0.70 - 1.04)	0.66	(0.56 - 0.78)	0.63	(0.48 - 0.83)

### Socioeconomic trends

**Figure 2:**
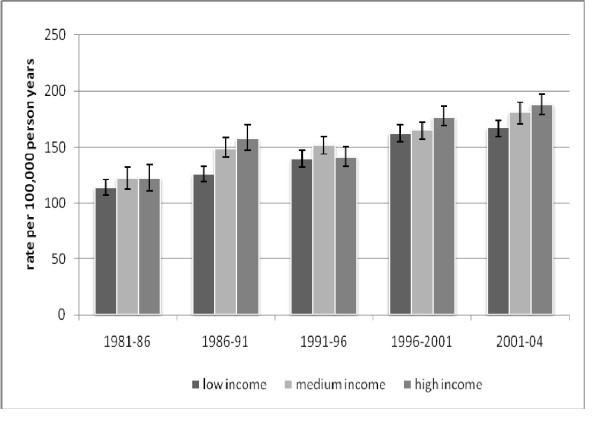
**Trends in age standardized incidence rates of breast cancer by time period and income tertile, age 25+**.

Figure 2 shows age and ethnicity standardised breast cancer incidence rates for low, medium and high income women for each cohort. A gradient with income is clearly seen in 1986-91, 1996-01 and 2001-04, with high income women having consistently higher rates, medium income women intermediate rates, and low income women the lowest rates. Over time, rates increase by about 50% in all income groups.

Table 4 shows the age and ethnicity standardised rates for each income group, and the relative and absolute differences of medium and low compared to high income women for each cohort. The differences were relatively stable over time, with no definite increase or decrease in the differences between income groups over the time period studied. Overall low income women had rates approximately 10% lower than high income women.

**Table 4:** Age and ethnicity standardised breast cancer rates, rate ratios (SRR), and rate differences (SRD) by income, age 25+

	Cohort	SR	95% CI	SRR	95% CI	SRD	95% CI
**Income**							
**High**	1981-86	122	(111 - 134)	1		0	
	1986-91	158	(147 - 170)	1		0	
	1991-96	141	(133 - 150)	1		0	
	1996-01	177	(169 - 186)	1		0	
	2001-04	188	(179 - 197)	1		0	
	% Change		54%				
	*P (Trend)*		*0.04*				
	Pooled	156	(151 - 160)				
							
**Medium**	1981-86	122	(112 - 132)	0.99	(0.88 - 1.13)	-0.7	(-16 - 14)
	1986-91	149	(141 - 158)	0.94	(0.86 - 1.03)	-8.8	(-23 - 5.4)
	1991-96	152	(144 - 159)	1.07	(0.99 - 1.16)	10	(-1.3 - 22)
	1996-01	165	(157 - 172)	0.93	(0.87 - 0.99)	-13	(-25 - -1.2)
	2001-04	181	(171 - 190)	0.96	(0.90 - 1.03)	-7.5	(-20 - 5.4)
	% Change		48%				
	*P (Trend)*		< .*01*		*0.67*		*0.59*
	Pooled	152	(148 - 156)	0.98	(0.94 - 1.01)	-3.7	(-9.7 - 2.2)
							
**Low**	1981-86	114	(107 - 121)	0.93	(0.83 - 1.04)	-8.8	(-22 - 4.6)
	1986-91	126	(119 - 133)	0.80	(0.73 - 0.87)	-32	(-46 - -19)
	1991-96	139	(132 - 147)	0.99	(0.91 - 1.07)	-1.9	(-13 - 9.0)
	1996-01	162	(155 - 170)	0.91	(0.86 - 0.98)	-15	(-27 - -3.6)
	2001-04	167	(159 - 174)	0.89	(0.83 - 0.95)	-22	(-34 - -9.5)
	% Change		47%				
	*P (Trend)*		< .*01*		*0.94*		*0.83*
	Pooled	140	(137 - 143)	0.90	(0.87 - 0.93)	-16	(-21 - -10)

*p test is test for linear trend

## Discussion

This study demonstrates a gradient of increasing breast cancer incidence with increasing income, stable over the time period studied. By ethnicity, lower breast cancer incidence rates were found amongst Pacific and Asian women compared to European/other women. Young Pacific women, however, had relatively high rates. Higher and more rapidly increasing rates were found amongst Māori women across all age groups.

The relatively high rates found amongst women of higher income levels are consistent with international patterns, and with local information about the distribution of known risk factors. The patterns seen in Pacific and Asian women are also consistent with what is known about the distribution of risk factors. High rates amongst Māori women are, however, a surprising finding given what is known about reproductive patterns amongst Māori and the distribution of other known risk factors. Investigation of the high and steeply increasing rates amongst Māori women is important for reducing the burden of disease in this group, but also may provide important clues to improved understanding of the aetiology of breast cancer; we offer some potential explanations to test below.

### Ethnic differences and trends

Breast cancer rates in European women in New Zealand, which rose from 114 to 170/100,000 women per year between 1981-86 and 2001-04 (for women 25+), are comparable to breast cancer rates in European women in other developed countries. For example, in the US non-Hispanic white women have an annual age standardised rate of 189/100,000 for women 25+, (based on 14 SEER registries, 1998-2002; using the same WHO World Standard) [2]. In Scotland women 25+ have an annual age standardised rate of 152/100,000 (1998-2002; using WHO World Standard) [2].

The incidence pattern for breast cancer amongst Pacific women is consistent with our knowledge of breast cancer risk factor distribution in this group. Pacific women are more likely than European women to have children, to commence child bearing earlier and to have more children [37]. Lower alcohol consumption [38] will also contribute to the lower risk of breast cancer amongst Pacific women. The higher rates seen in younger Pacific women (Table 3) may be related to the cancer promoting effect of pregnancy. A similar pattern is seen in the US, with young black women having higher rates than young white women (and older black women having lower rates than older white women), which is thought to relate to earlier childbearing amongst black women [15]. Age at first birth data in New Zealand is only collected for "nuptial" births and is not reported by ethnic group. However based on age specific fertility rates [39] it is apparent that although Pacific women have similar early fertility rates to Māori women, both groups have age specific fertility rates more than double those of European women up to age 25, and so differences in early parity may explain the differences in breast cancer incidence seen between young Pacific and European women.

Rates of breast cancer amongst Asian women were consistently lower than those of European and Māori women. This "Asian" group is diverse both in terms of country of origin and time in New Zealand, and so is likely to be diverse in terms of reproductive patterns and other risk factors for breast cancer, making the results for Asian women hard to interpret. However despite small numbers, the relatively low rates seen are consistent with the low rates seen in Asian countries. For example IARC's Cancer Incidence in Five Continents IX reports age standardised rates per 100,000 for those aged 25+ of 52 in India, 67 in China, 45 in Korea, and 56 in Malaysia for the period 1998-2002 (based on available cancer registry data, standardised to WHO World Standard) [2]. The ASR of 126 per 100,000 (95% CI 110-142) found amongst New Zealand Asian women in 2001 - 2004 is considerably higher than rates in Asian countries, which is likely to reflect changes in risk factor distribution following migration.

The high incidence of breast cancer amongst Māori women is however not easily explained, as Māori women have a more favourable (known) risk factor profile than European women. Māori women tend to start having children younger and have more children compared to European women [37]. Māori women have lower rates of HT use [40], and similar rates of oral contraceptive use, alcohol consumption and physical activity compared to European women [38,41]. Screening is not contributing to the higher incidence seen, as Māori women have lower uptake of screening [42]. All of this risk factor information suggests that European/Other women should have higher rates than Māori, yet it is Māori who consistently do so. One partial explanation for the observed higher rates for Māori is post-menopausal obesity among Māori, however it is unlikely to be a full explanation for higher post-menopausal rates.

The timing of menarche has previously been postulated as a possible reason for the difference in breast cancer rates between Māori and European women [43]. However the evidence to support differences in the timing of menarche and menopause between Māori and non-Māori women is inconclusive and any differences in timing are small [24,40,44]. The prevalence of other postulated breast cancer risk factors such as shift work [45], exposure to particular environmental pollutants [46], or Vitamin D levels [47] may differ between ethnic groups but is unlikely to explain the large differences identified here.

Several recent studies have suggested that cigarette smoking initiation shortly after menarche and before first full term pregnancy may increase the risk of breast cancer [48-50]. Early initiation (mean age 11 in 2007) [51] and very high rates of tobacco use amongst young Maori women [52] may be important for breast cancer risk, although the effect of early initiation of smoking on breast cancer risk does not appear to be large enough to explain the patterns seen.

There is some evidence that there are differences in tumour biology between ethnic groups. For example, there are differences in tumour grade and estrogen and progesterone receptor (ER and PR) positivity between black, Hispanic and white women in the US [53]. In New Zealand, the potential of genetic differences between ethnic groups has not been thoroughly explored and findings in relation to receptor positivity status have been inconsistent [54,55]. Regardless, while differences in tumour biology may be important with respect to survival differences between women with breast cancer in different ethnic groups [56], it is unclear whether they are important for explaining differences in incidence. Moreover, such genetic differences will not account for rapid changes in rates over time in any group, and could only account for diverging ethnic group trends over time in the presence of some environmental exposure that is also changing over time and interacting with a varying genetic predisposition by ethnicity.

Indigenous Hawaiian women also have surprisingly high rates of breast cancer given their distribution of risk factors, and it has been postulated that genetic polymorphisms in the sex steroid and gonadotropin metabolism pathways, causing high endogenous hormone levels, are responsible for their high rates [58]. Dietary factors and high insulin-like growth factor levels are also mentioned as possible factors [57]. Indigenous Hawaiians also have higher breast density than European Hawaiians [58]. It is possible that there may be similarities between the drivers of breast cancer rates in Māori and indigenous Hawaiian women, but more research is needed to elucidate potential mechanisms in both groups. However it is not obvious why Māori and Hawaiian women would have such different breast cancer rates from other groups of Polynesian women from the Pacific.

The above discussion has been orientated mainly at trying to explain differences between ethnic groups' rates on average, but our study also finds a more rapidly increasing Māori breast cancer rate than European/Other. (The Asian rate also increased rapidly, but was prone to greater statistical imprecision - hence we focus on just Māori and European/Other trends.) This divergence over time may be due to changes in the factors which are driving the underlying differences between Māori and European/Other rates (although as noted above these factors are currently unknown). Alternatively, this change may be due to changes in the distribution of established risk factors amongst the Māori population, overlying the already higher risk for Māori. For example there is some evidence that Māori fertility rates are dropping more quickly than among European women, with a convergence on similar fertility patterns [37,59]. Rapid changes in postmenopausal obesity rates amongst Māori women may also be an important factor in this change.

Finally, it is important to note that breast cancer mortality differences between Māori and non-Māori remain more significant [26] and are more likely to be amenable to intervention than incidence differences, and so should remain a central concern of researchers and policy makers.

### Socioeconomic trends

The pattern of higher breast cancer rates amongst women of higher socioeconomic status seen here is consistent with that found internationally [60-62], and is likely to be primarily due to differences in reproductive behaviour. Socioeconomic differences in reproductive behaviour, including differences in parity, age at first birth, childlessness, and the duration of breast feeding, are seen in almost all settings internationally [12]. In New Zealand there are significant fertility differentials by level of education, with women with the highest level of education having the fewest children, and this difference is greatest in women born more recently indicating an increasing socioeconomic differentiation in reproductive behaviour [63]. It is possible that over time this will result in widening of differentials in breast cancer rates between socioeconomic groups in New Zealand. Screening uptake and HT use have also been socioeconomically patterned, with higher uptake amongst better off women [64], and this will contribute to the difference in incidence seen. HT use in New Zealand declined markedly following the publication of the results of the Women's Health Initiative trial in 2002 [65], and this is likely to have reduced the difference in the last time period by socioeconomic status.

### Strengths and Limitations

This study is a series of five short-duration cohort studies of the entire New Zealand population, followed up for breast cancer over a 24 year period. By using data from the entire New Zealand (census night) population as well as data from New Zealand's population based cancer registry, it was possible to overcome numerator/denominator bias and misclassification of ethnicity and socioeconomic group. Self identified ethnic affiliation from census forms was used to define the numerator and denominator populations, and therefore produce more reliable estimates of differences between ethnic groups. Individual measures of socioeconomic position were also able to be used rather than relying on area-based measures. Considering trends over 24 years also provides a good basis for understanding evolving differences between population groups.

However the study is not without limitations. Statistical imprecision becomes a problem when stratifying by ethnicity or socioeconomic position. This is particular problem for the Asian and Pacific groups where numbers are small. It was not possible to link all cancer records back to the census, and so weighting for linkage bias was undertaken; we are confident that this will have eliminated most bias due to misclassification of outcome. The Cancer Registry Act, which came into effect in 1995, required mandatory registration of all cancers, and there may have been a small artefactual increase in breast cancer registrations following its introduction, but this is not thought to have a had a major effect on breast cancer rates and probably impacted ethnic groups and income groups in a similar manner.

Household income is presented as an indicator of individual socioeconomic status. While education measures socioeconomic status closer to the time of reproductive choices which are important for breast cancer risk, more proximal exposures such as post menopausal obesity, screening, and HT use are also important for breast cancer risk and so both measures are relevant. Education also lacks discriminatory power in older women in the earlier cohorts, as the majority of these women fall into the category of no qualifications. As similar results were found for both education and household income a decision was made to only present the results for household income in this paper.

Finally, the lack of good information about risk factor distribution in the population, particularly in the relevant time period, makes it difficult to fully interpret the differences found between ethnic and socioeconomic groups; rather, these breast cancer rates over time might provide clues about unmeasured risk factors back in time.

## Conclusions

Patterns of breast cancer incidence in Pacific women and between socioeconomic groups are probably explicable in terms of the known risk factors for breast cancer. However no straightforward explanation for the relatively high incidence amongst Māori is apparent. Biological explanations put forward for unexpectedly high rates amongst Hawaiian women may provide a clue, but little is known about biological differences in breast tumours or risk factors between Māori and non-Māori and much more work needs to be done in this area. Even if underlying biological or genetic differences explain some of the difference in rates, the faster increase in rates amongst Māori women may in fact be the result of changes in risk factor distribution.

Further research should focus on the role of known and new risk factors and the biological characteristics of breast tumours as determinants of the pattern of breast cancer incidence amongst Māori women. It may be that the currently inexplicable differences between Māori and European/Other women at any one point in time, and diverging trends over time, provide a chance for research that can provide new insights into the aetiology of breast cancer. Moreover, such insights may contribute to reducing the burden of breast cancer amongst Māori women.

## Competing interests

The authors declare that they have no competing interests.

## Authors' contributions

CS and TB were responsible for the inception and design of the study. JA participated in the design of the study and was responsible for the analysis of data. RC was responsible for the interpretation of data and drafted the manuscript. CS, DS and TB provided comments on drafts of the manuscript. All authors read and approved the final manuscript.

## Pre-publication history

The pre-publication history for this paper can be accessed here:

http://www.biomedcentral.com/1471-2407/10/674/prepub
